# Virtual Tumor Mapping: A New Standard for Surgeon–Pathologist Collaboration in Treating Oral Squamous Cell Carcinoma

**DOI:** 10.3390/cancers16223761

**Published:** 2024-11-07

**Authors:** Adam Michcik, Maksym Jopek, Rafał Pęksa, Piotr Choma, Łukasz Garbacewicz, Adam Polcyn, Tomasz Wach, Maciej Sikora, Barbara Drogoszewska

**Affiliations:** 1 Department of Maxillofacial Surgery, Medical University of Gdansk, Mariana Smoluchowskiego 17, 80-214 Gdansk, Poland; pchoma@uck.gda.pl (P.C.); lgarbacewicz@gumed.edu.pl (Ł.G.); adampolcyn@gumed.edu.pl (A.P.); drog@gumed.edu.pl (B.D.); 2 Laboratory of Translational Oncology, Intercollegiate Faculty of Biotechnology of the University of Gdańsk and the Medical University of Gdańsk, Dębinki 1, 80-211 Gdańsk, Poland; maksym.jopek@gumed.edu.pl; 3 Centre of Biostatistics and Bioinformatics, Medical University of Gdansk, Mariana Smoluchowskiego 17, 80-214 Gdansk, Poland; 4 Department of Pathomorphology, Medical University of Gdańsk, Mariana Smoluchowskiego 17, 80-214 Gdansk, Poland; rafal.peksa@gumed.edu.pl; 5 Department of Maxillofacial Surgery, Medical University of Lodz, Zeromskiego 113, 90-549 Lodz, Poland; tomasz.wach@umed.lodz.pl; 6 National Medical Institute of the Ministry of Interior and Administration, Wołoska 137 Str., 02-507 Warsaw, Poland; sikora-maciej@wp.pl; 7 Department of Maxillofacial Surgery, Hospital of the Ministry of Interior, Wojska Polskiego 51, 25-375 Kielce, Poland; 8 Department of Biochemistry and Medical Chemistry, Pomeranian Medical University, Powstanców Wielkopolskich 72, 70-111 Szczecin, Poland

**Keywords:** cancer imaging, oral squamous cell carcinoma, scanning 3D, tumor mapping, virtual communication, surgeon–pathologist

## Abstract

**Background:** The histopathological assessment is critical in the comprehensive treatment process for patients diagnosed with oral squamous cell carcinoma (OSCC). A detailed and precise specimen characterization is essential to facilitate effective surgeon–pathologist communication. **Methods:** In response to this need, a user-friendly virtual communication protocol utilizing a 3D scanner has been developed. This study involved 50 patients with OSCC, whose resected tumors were directly scanned in the operating room and subsequently annotated and characterized using available software. **Results:** The direct application of annotations and descriptions onto the virtual tumor specimens significantly enhanced the quantity and accuracy of information conveyed to the pathologist. **Conclusions:** The proposed solution’s repeatability and standardized approach make integration into routine clinical practice feasible, thereby establishing a potential new standard in the field.

## Simple Summary

Therapeutic decisions made during the treatment of patients with oral squamous cell carcinomas (OSCCs) significantly impact the overall course of the disease. A critical stage in the treatment process is the accurate histopathological assessment of excised tumors. The precision of the specimen description and the comprehensiveness of the information provided to the pathologist are of paramount importance. To address this, a multidisciplinary team comprising a maxillofacial surgeon, a pathologist, and an engineer was assembled. The team’s objective was to devise a user-friendly protocol for virtual communication between the surgeon and the pathologist. Leveraging available software and a 3D scanner, a virtual communication protocol was formulated, enabling the generation of virtual images of excised tumors. Moreover, a methodology for applying annotations and marking specific areas (e.g., margins) directly on the tumor surfaces was developed. Consequently, this approach has afforded the team extensive capabilities for effectively conveying and archiving precise information.

## 1. Introduction

Oral cancers are 90% squamous cell carcinomas (OSSCs) [1]. Their clinical course depends on many factors, including location, type of growth, and size of the tumor [2], and it is difficult to predict. The therapeutic process is complex and influenced by many variables. Additionally, it is important to note that 48% of the survival rates for 5-year patients with OSCC remain unsatisfactory [3]. Oral carcinogenesis is a multifactorial process that occurs when epithelial cells are affected by genetic alterations such as TP53, EGFR (epidermal growth factor receptor), CDKN2A (cyclin-dependent kinase inhibitor 2a), NOTCH1 (Notch homolog 1 genes are translocation-associated), and STAT3 (signal transducer and activator of transcription 3) [4]. Clinically, the neoplasm develops de novo or based on a precancerous lesion [5,6,7]. It is primarily characterized by an endophytic growth pattern that infiltrates surrounding tissues [8,9]. One of the first symptoms, especially in the case of locations within the tongue (TC) and floor of the mouth (FOM), is pain [10], followed by dysphagia and odynophagia. In a relatively short time, the tongue becomes immobilized and gibberish speech is formed. Its characteristic feature is the formation of nodal metastases to the neck lymphatic system, unilateral or bilateral [11] and, less frequently, distant metastases [12]. Analyses of prognostic factors available in the literature [2,13] show how many of them are of great clinical significance.

However, to achieve therapeutic success and increase the chance of 5-year survival, it is necessary to search for new solutions that will increase accuracy at each stage of treatment and contribute to making the right decisions throughout the therapeutic process.

One of the key stages is the histopathological assessment of the excised tumor and the neck lymphatic system [14]. It determines further therapeutic decisions: possible surgical widening of the margins and the decision to observe, start, or discontinue radiotherapy. According to my experience and that of other surgeons, excised oral OSCCs are most often spatial structures with an irregular surface and shape. For this reason, as well as due to the complex structure and the presence of many anatomical structures in a small area, pathological assessment of oral cavity tumors is often a challenge for the pathologist. Until now, the surgeon had no other option to communicate with the pathologist than the traditional annotation or marking of the specimen, e.g., with a thread or bead. Following the excision of the tumor, the pathologist permanently loses the ability to conduct a comprehensive assessment of the entire specimen. At present, there is an increasing trend in the medical field toward the integration of computer-aided diagnostic tools and virtual reality technologies.

3D imaging techniques are widely used in various fields, particularly in radiology. Creating three-dimensional images based on CT, MRI, or volumetric CT imaging is now the standard [15,16]. The use of 3D images created based on imaging tests is used in oncological surgery, including, among others, urological surgery [17], pancreatic [18], breast [19], and finally head and neck surgery [20,21]. With the advancement in virtual imaging techniques, high-resolution surface scanners, originally intended for technical applications, have begun to be integrated into medical practice alongside traditional imaging tests. These devices provide a more detailed object description but also allow for color capture in the form of 3D model texture, which is not available in the case of MRI. This provides an additional layer of information that is possibly of high value to a physician. The available works describe the use of the scanner to assess patients with OSCC during radiotherapy [22], or preparation for this type of treatment [23,24]. Only a limited number of publications exist that detail the 3D scanning of excised oral tumors [25,26,27]. In our study, we utilized a specialized scanning method to conduct tumor analyses. It is imperative to illustrate the efficacy of our method in generating virtual tumor images. These images serve to enhance the quality of medical documentation significantly. This decision was driven by the desire to explore the potential of this technology. The project aimed to create a user-friendly 3D image-based annotating methodology, allowing for precise descriptions and the direct application of markings to the virtual tumor, which would serve better surgeon–pathologist communication.

## 2. Materials and Methods

The analysis included 50 patients with oral tumors operated on at the Maxillofacial Surgery Department of the Medical University of Gdańsk in 2023–2024. All participants signed a written consent. Patients with OSCC T1 to T4b located in the floor of the mouth (FOM, n = 25), tongue (TC, n = 16), retromolar triangle (RMT, n = 5), and maxilla (MT, n = 4) were enrolled in this study. All patients were over 18 years of age (mean age 68.4). Participants in this study were not disqualified based on the presence of general diseases, such as metabolic, cardiovascular, autoimmune, or neurological conditions. In the case of qualification for surgical treatment, these general diseases did not impact the procedure for tumor excision. The study cohort description is provided in Table 1.

### 2.1. Tumor 3D Sample Acquisition

Each of the patients, apart from the excision of the primary tumor, underwent neck dissection of appropriate scope and, in most cases, reconstruction with the use of free flaps. There was no exclusion criterion for the scanned tumors, although we had problems with accurately scanning the extensive maxilla tumors (due to numerous arcades and difficulties in setting it up on a tripod). For this reason, 2 patients were not included in this study because we were unable to obtain full scan images. In the event of a tumor tearing during dissection or other damage, we scanned all parts (except small fragments), and then during processing in Blender (https://www.blender.org/), we combined them virtually to recreate an image of the entire tumor or mapped them separately to provide the pathologist with as much information as possible.

**Figure 1:**
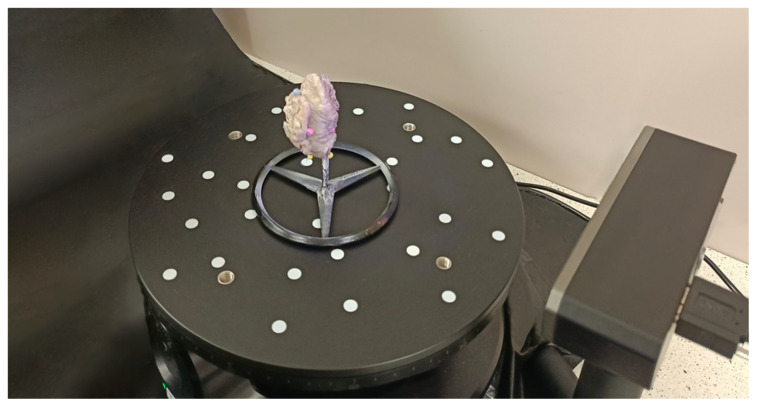
Photo of the scanner and the turntable with the constructed stand.

The tumors were scanned immediately after excision in the operating room, and then they were placed in a formalin bath following the protocol. The scanning procedure consisted of four steps. First, the tumor was contrasted with a matte spray designed for Aesub Blue 3D scanners (composition: propane, ethanol, tricyclo decane, hydrocarbons, C6-C7, iso-alkanes, cyclics, <5% n-hexane, n-hexane), which evaporates from the object in a short time without leaving a trace on it [28]. Tumor matting does not affect the histopathological assessment of the specimen but increases the accuracy of scanning. The residual preparation that had not sufficiently evaporated was rinsed with a 0.9% saline solution. Secondly, the tumor was mounted on a dedicated rotating table with a custom-designed stand. Its purpose was to allow the scanner to cover a broader part of the tumor (Figure 1).

**Figure 2:**
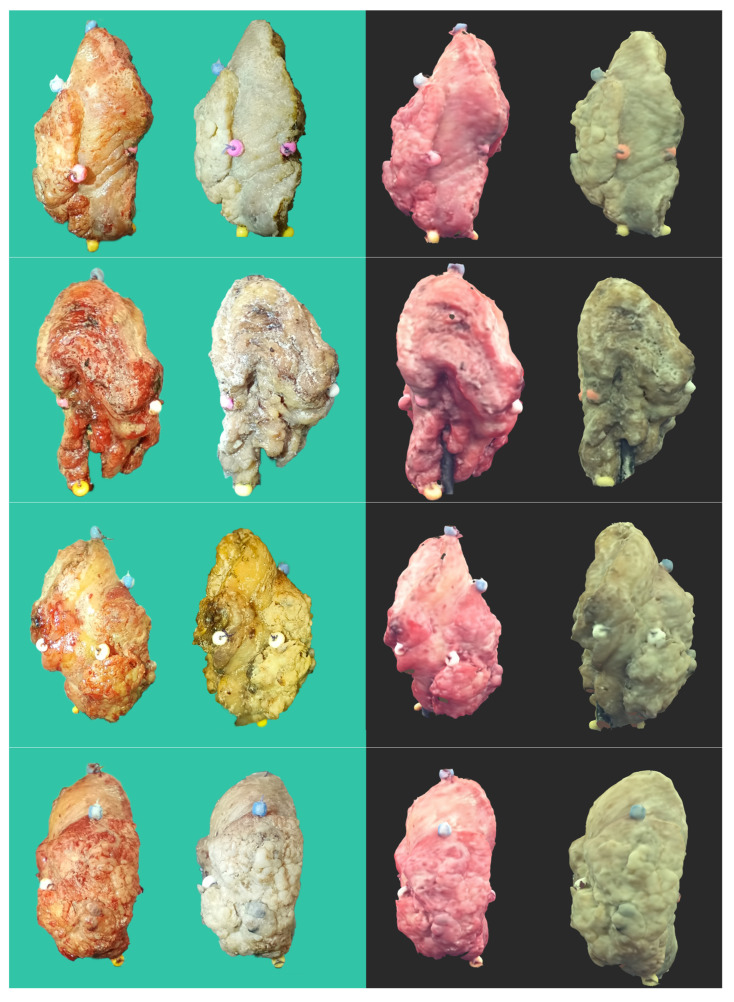
Tongue OSCC. The material provides a comprehensive view of the tumor on all sides, highlighting the precision of the specimens obtained through the 3D scanning technique before and after undergoing formalin fixation. Green background—photos of the tumor after excision and after formalin fixation. Black background—images of tumor scans after excision and after formalin fixation.

To further increase the accuracy of scanning, a black photographic background surrounding the scanner was used. Next, scanning was performed using a 3D scanner: Revopoint MINI—Dual-axis Turntable Package (Shenzhen, China) adapted to scan small objects, with an accuracy of 0.02 mm at the speed of 10 frames per second. The scanner was chosen for its ultra-high resolution and ambient light resistance (Figure 2).

**Figure 3:**
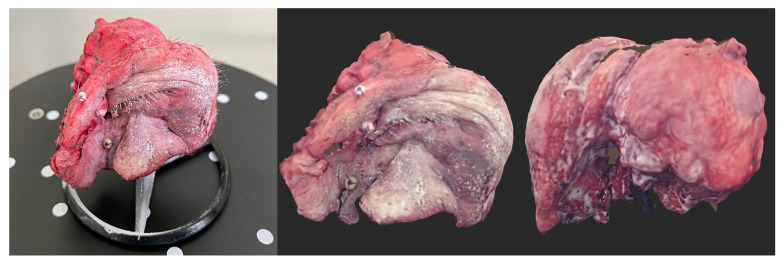
Orbital and maxilla tumor. The image on the left side is of the tumor and the stand before scanning. The image on the right is of the tumor, created with a 3D scanner.

Lastly, the obtained virtual objects in the form of STL (triangulated representation of the surface geometry in three-dimensional space) were processed in the Revo Scan 5 (https://global.revopoint3d.com) program (among other things, the environmental impurities were removed, and the surface of the tumor object was sharpened). The processed 3D images were then saved as OBJ files, along with the models’ textures saved as JPGs (Figure 3).

### 2.2. Image Annotation

**Figure 4:**
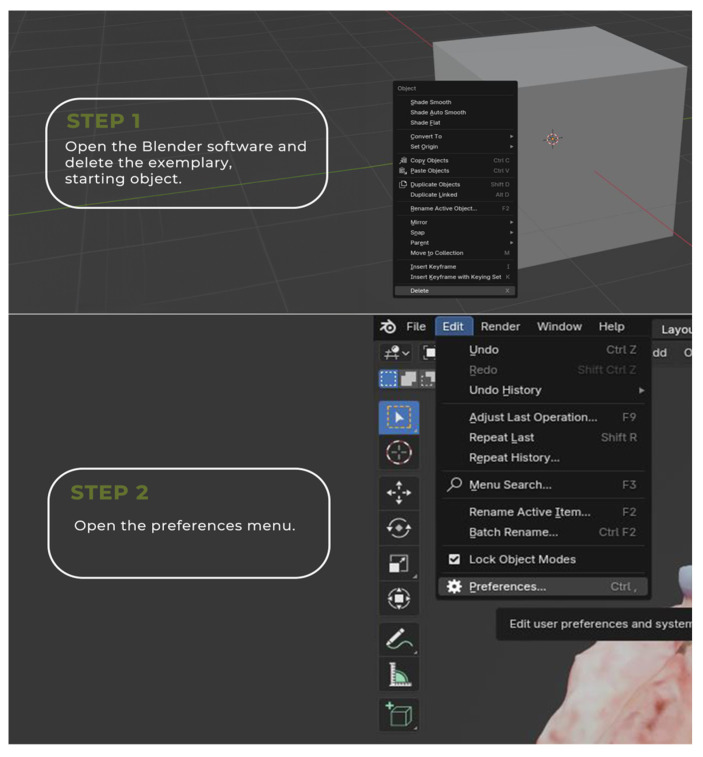

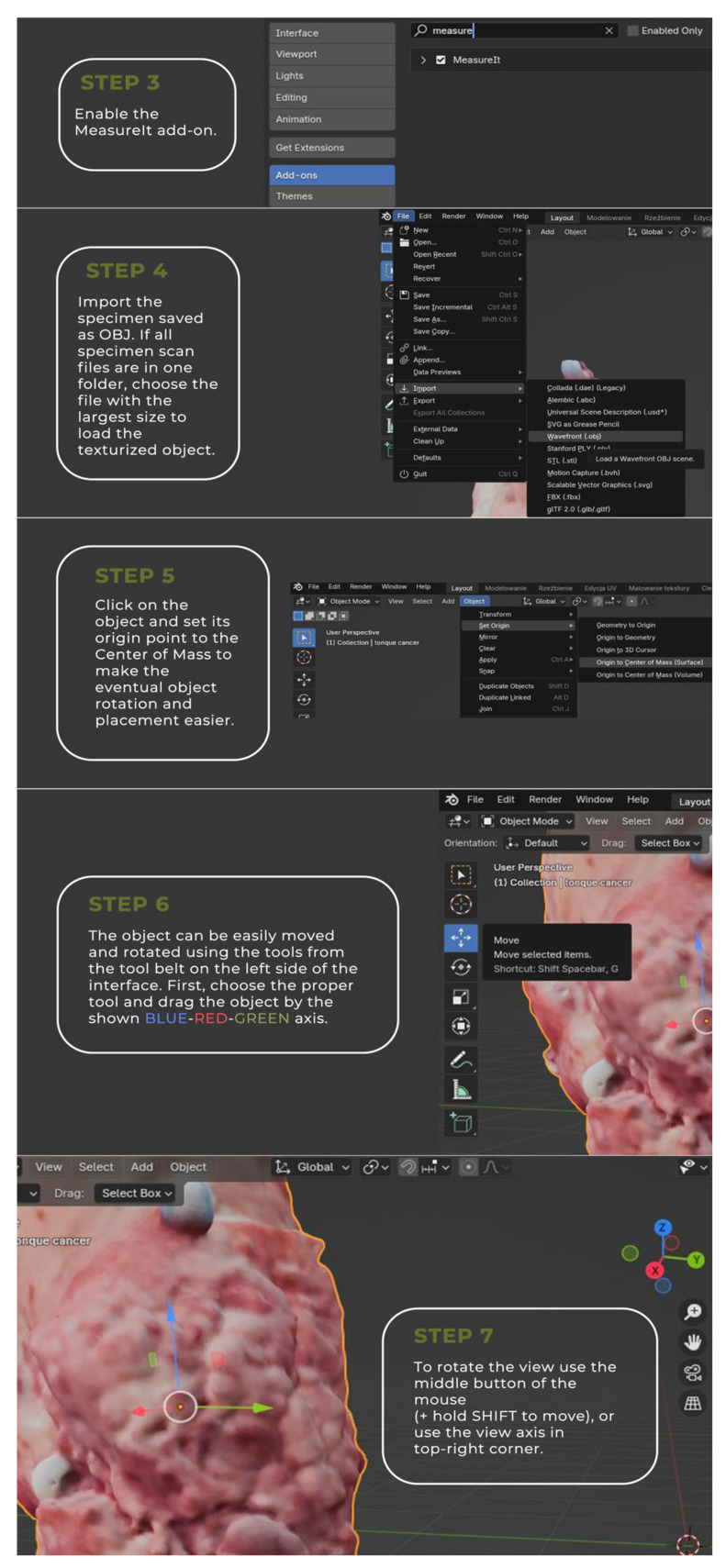
Comprehensive instruction for the virtual annotation, mapping, and measurements of the tumor.

**Figure 5:**
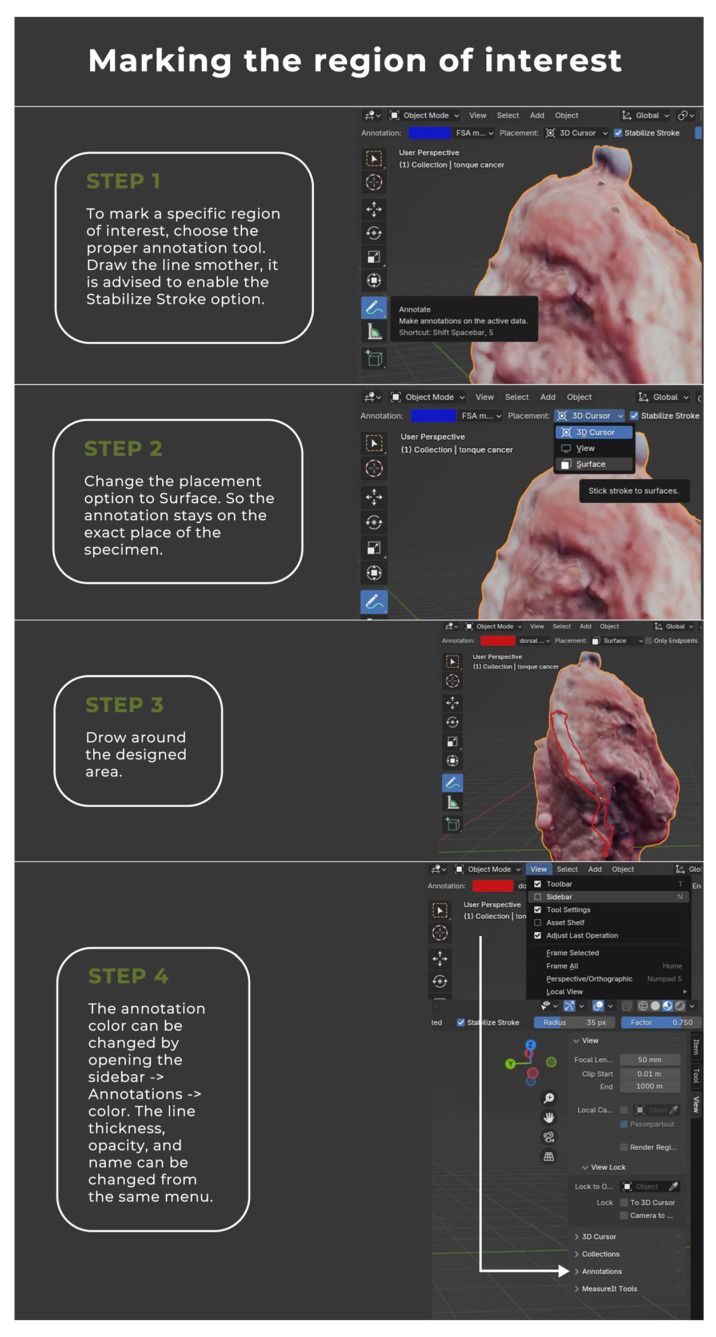

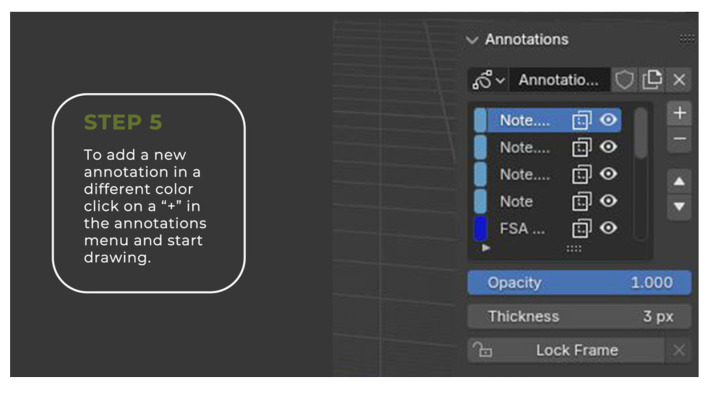
Comprehensive instruction for the virtual annotation, mapping, and measurements of the tumor.

**Figure 6:**
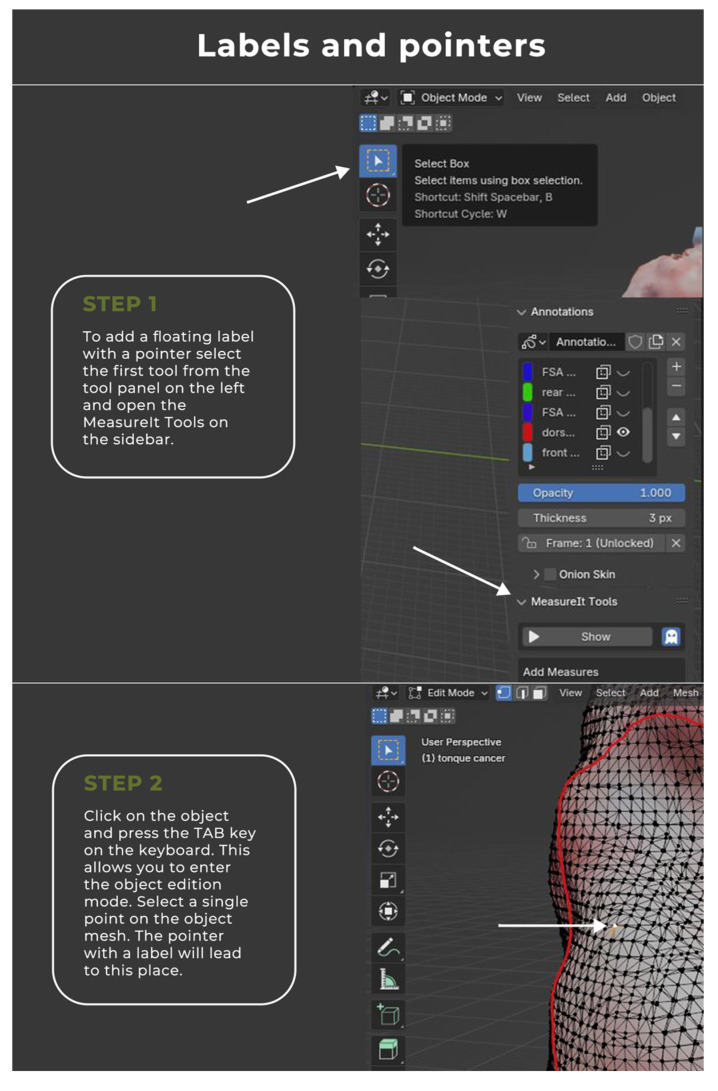

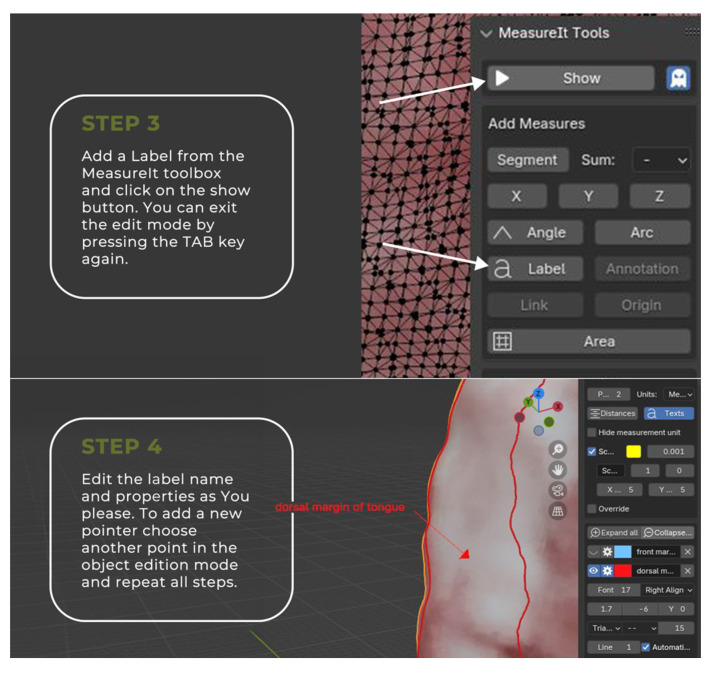
Comprehensive instruction for the virtual annotation, mapping, and measurements of the tumor.

**Figure 7:**
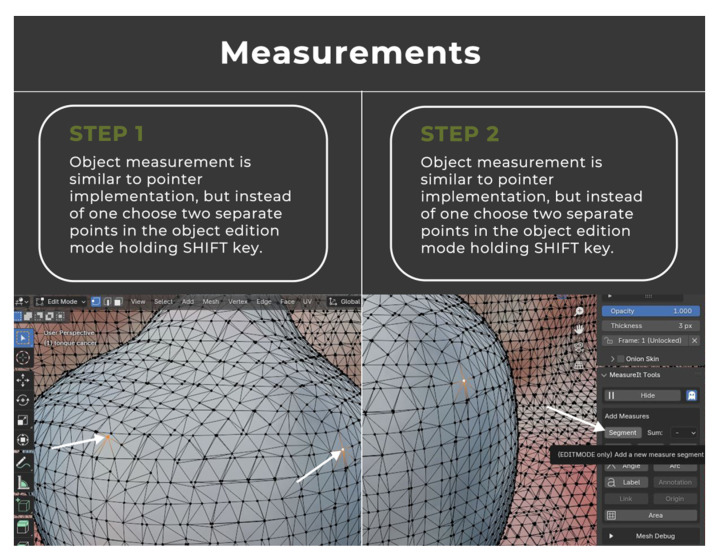

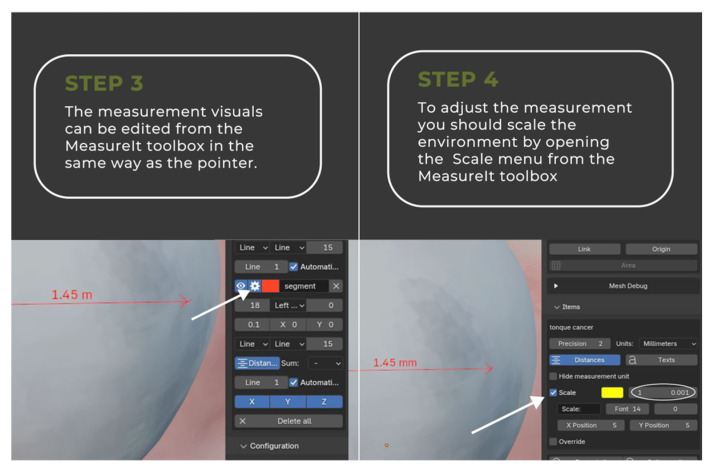
Comprehensive instruction for the virtual annotation, mapping, and measurements of the tumor.

The image annotation process was performed in Blender 4.1.1 open-source software (www.blender.org, accessed on 25 September 2023) with a pre-installed Measure It plugin. The software was chosen for its availability, open-source (free of charge), and easy-to-operate interface. It was originally created as a 3D modeling system for digital artists, but over the years its uses have expanded to movie editing and motion capture technology. This program made it possible to apply annotations directly on the surface of 3D objects, create floating pointers with notes, and measure the object upon earlier environment scale calibration (Figure 4, Figure 5, Figure 6 and Figure 7).

**Figure 8:**
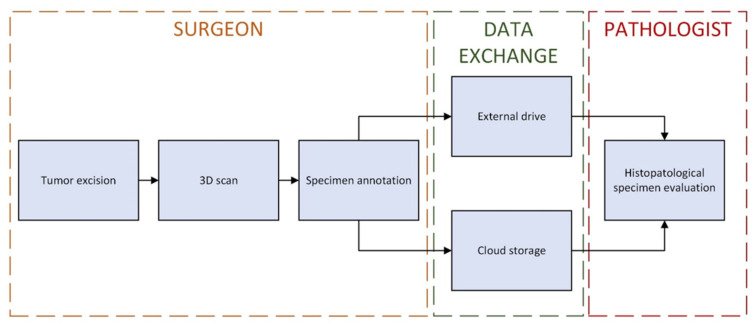
Simplified 3D image annotation and data exchange process.

The annotated blender 3D images can be easily exchanged between various personas via data storage or a cloud-based system. The usage of locally available technologies is advised to eliminate data security problems. The simplified process has been visualized in Figure 8.

## 3. Results

**Figure 9:**
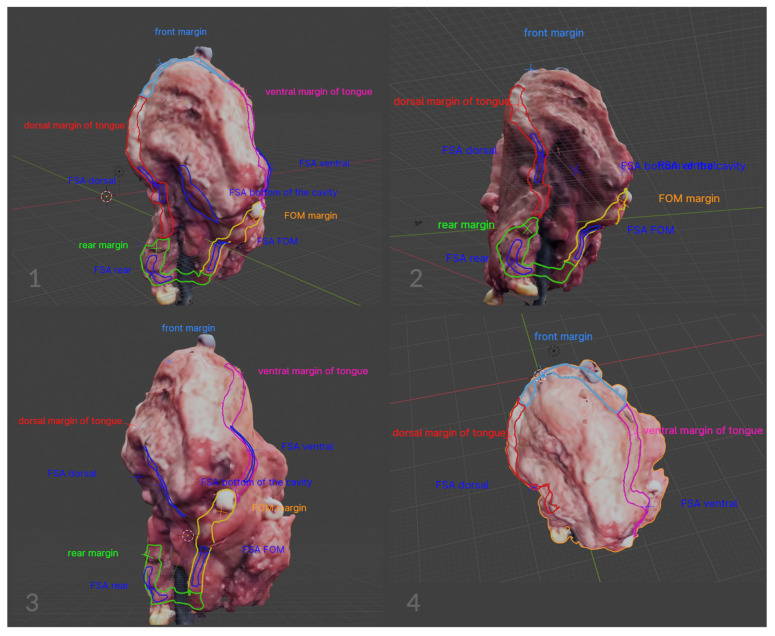
Tongue tumor mapping scheme. The virtual image facilitates the comprehensive evaluation of all tumor surfaces. Flat-labeled margins and frozen section analysis (FSA) sampling locations are visible. The figures demonstrate the mapping capabilities directly on the surface of a virtual tumor. In the course of a virtual tumor assessment, it is feasible to temporarily deactivate specific determinations. This action significantly increases the clarity of the image.

Using the proposed methodology, each of the 50 qualified tumors was described in detail by marking the margins and their boundaries in a planar way; the orientation of the tumor was determined, as well as possible comments that could help in the subsequent histopathological assessment of the preparation. In addition, the places on the tumor were marked directly in the operating room, from which margins were taken for the frozen section analysis. The margins were taken from the excised specimen, and the corresponding areas were marked on the scanned tumor, which significantly increased the amount of information and the accuracy of the specimen description (Figure 9).

The files were then exported to the cloud, from where they were downloaded by the pathologist during the traditional histopathological assessment after formalin fixation. An analysis of scanning tumors after formalin fixation was also performed—no significant changes in the shape of the tumors were observed. This allowed the pathologist to rely on the scan and description of the fresh specimen during the histopathological evaluation of the formalin specimen. In addition, sewn plastic markers with letters or numbers increased the amount of information provided to the pathologist and the orientation of the tumor itself. Three-dimensional scanning provides a detailed and comprehensive view of the tumor from all angles, allowing pathologists to examine the specimen more thoroughly than with traditional 2D images. The precise measurements obtained from 3D scans help accurately assess margins, leading to more accurate completeness of surgical excision. Furthermore, 3D scans create a permanent digital record of the specimen, which can be reviewed multiple times and compared with future scans, providing continuity in patient care and can be shared easily with other medical professionals, enhancing multidisciplinary discussions and collaborative decision-making. Moreover, digital scans reduce the need for physical handling of specimens, which can be especially beneficial for fragile or small samples, preserving their integrity, and can also be used as valuable educational tools for training pathologists, surgeons, and medical students, providing a realistic and interactive way to study tumor anatomy and pathology (Table 2).

## 4. Discussion

In the present study, a protocol for virtual surgeon–pathologist communication was developed. The method’s reproducibility, schematic nature, and relatively short scanning time (5 min on average, depending on variables like size and complexity) make virtual mapping of resected tumors a potential standard stage of the surgical procedure. This study took into account the opinions of pathologists and surgeons from the team and demonstrated the method’s superiority over the traditional description of the excised tumor. The method significantly increased the amount of information provided to the pathologist and its accuracy. Future studies should also explore scanning smaller samples. In this case, the scanning protocol must be modified. The description of each of the 50 tumors was meticulously conducted following a predefined scheme in collaboration with the pathologist. According to the pathologist, the ability to review the virtual descriptions throughout the entire histopathological assessment process proved to be invaluable. For cases requiring reoperation, 3D scans of the excised tumors can provide critical information that aids in planning subsequent surgical procedures more precisely and offer a robust method for documenting and following up on cases, providing valuable data for clinical research and studies on treatment outcomes and tumor behavior. In our study, the retrospective analysis of 50 qualified patients during oncology consultations influenced therapeutic decisions in five cases, leading to the implementation of radiotherapy. Additionally, 3D scans can be integrated with other imaging modalities like MRI or CT, providing a more comprehensive understanding of the tumor’s characteristics and relationship with surrounding tissues. Despite the limited existing literature, we have devised a scanning method that allows comprehensive imaging of tumors. Koivuholm et al. encountered similar challenges and proposed the utilization of a tumor support mesh during the scan [27]. This method of scanning proved to be difficult for everyday and long-term use due to the type of construction. After many (about 100) attempts to scan liver and turkey hearts and tumors traditionally on a turntable, we found the method inadequate. Placing the specimen on the table did not result in a complete spatial image of the tumor, and efforts to combine two scanned images of the same tumor, even with sewn-on markers, were unsuccessful. This was attributed to the irregular and complex shape of the slides and the slightest movements of the markers when changing the side. We consulted with a Biomedical Engineering specialist from Gdańsk University of Technology to discuss the methodology of combining the scanned objects. The scanning method on a specimen rotation table was modified by building a special tripod with bumps. The advantage of this modified method is its simplicity, repeatability, and longevity of the stand made of recycled metal. This study’s second objective was to generate virtual images of post-resection defects within the oral cavity using an intraoral 3D dental scanner. Creating detailed images of the defect would be invaluable for accurately describing histopathological preparations and would be particularly useful in cases requiring reoperation, such as incomplete excision of a tumor or widening of excision margins. Despite our efforts and software adjustments, we encountered challenges using the intraoral 3D scanner Shining 3D Aoralscan 3 (Hangzhou, China) to consistently image defects, as these dental scanners are primarily designed for scanning hard tissues like teeth. However, we believe that advancements in 3D imaging techniques will make this method feasible in the future. Our clinic’s team remains committed to exploring new solutions for this issue. Histopathological assessment of tumors represents a critical stage in the overall treatment process. As surgical techniques continue to advance, the methodology for evaluating specimens by pathologists has undergone gradual modifications. One widely utilized standard in resection operations for oral squamous cell carcinoma (OSCC) involves the use of intraoperative margin assessment [29]. The findings from a study conducted by Eugenie Du et al. revealed that 4.3% of the frozen section samples exhibited positive margins, while 17.8% had close margins that were not identified during the ad hoc study [29]. These results underscore the significance of detailed visualization and description of excised tumors. The proposed protocol by Aaboubout Y advocates for specimen mapping through the use of sewn-on markers to enhance the likelihood of achieving radical resection [30]. However, the transmission of tumor information in a traditional form led to the pathologist losing the overall image of the specimen after it was cut. In response, a mapping scheme was developed following consultations with pathologists to graphically present margins as planes and determine their exact range. The scheme also involved applying margins to the fresh specimen as collection site areas, as well as marking the edges of the specimen that are not margins but may pose challenges during histopathological assessment. For instance, in cases of maxilla cancer resection, fragments of the maxillary sinus wall or nasal mucosa (bordering on empty air space) may be encountered. Similarly, in cases of tongue cancer, the lateral margin constituting the tumor is invariably positive. The ability to refer to a virtual image of the tumor in such cases provides considerable convenience for pathologists. The graphic representation directly applied to the tumor presents a significant advancement in the communication between surgeons and pathologists. It is anticipated that this approach should become the gold standard in oncological surgery, offering an undeniable stride in improving communication and understanding between the surgical and pathological teams. It is important to emphasize that the proposed scheme is universally applicable and can be utilized not only by head and neck surgeons but also by oncological surgeons operating in various other anatomical regions. The methodology necessitates comprehensive training for both the surgeon and the pathologist in the utilization of 3D software, as well as for the operating room personnel in the proper use of the 3D scanner. Procuring high-quality scanning equipment is crucial for clinics, and fortunately, accessibility to such equipment is not a significant challenge in contemporary times due to its affordability.

## 5. Conclusions

The digitalization of medicine is an inevitable trend, and recent technological advancements have ushered in new possibilities that were previously unattainable. For instance, the widespread availability of devices such as 3D scanners, initially developed for technical purposes and prohibitively expensive, has revolutionized various fields, including medicine. This has particularly impacted the realm of maxillofacial surgery, where challenges in treating patients with oral squamous cell carcinoma (OSCC) are encountered regularly. Enhancing the precision and volume of information available during histopathological examinations is crucial, as it directly influences subsequent therapeutic strategies. The development of a standardized and user-friendly virtual imaging protocol for excised OSCCs has marked a significant milestone in improving communication between surgeons and pathologists. This protocol enables precise marking, measurement, and description of tumors, thereby substantially augmenting the information provided to pathologists. From the perspective of pathologists, the virtual representation of the tumor not only allows for ongoing analysis but also retrospective evaluations, which can be valuable during oncology consultations. High-quality 3D scans can be shared with remote pathologists or experts, enabling telepathology consultations and second opinions without the need to physically transport specimens. The outlined procedure in the manuscript is straightforward, reproducible, and can be adopted by other medical centers. At present, this protocol has been established as standard practice in our clinic, and it is implemented for every patient undergoing surgery for OSCC.

## Figures and Tables

**Table 1:** Characteristics of the study group, 8th TNM classification used, legends: TC—tongue cancer, FOM—floor of the mouth, RMT—retromolar triangle, MT—maxilla tumor, TPS—tumor primary site, G—grading, pT—tumor size, DOI—depth of invasion.

Characteristic		N	%
Gender	male	36	72
	female	14	28
Age	50th	6	12
	60th	16	32
	70th	18	36
	80th	10	20
TPS	TC	16	32
	FOM	25	50
	RMT	5	10
	MT	4	8
G	G1	12	24
	G2	26	52
	G3	12	24
pT	pT1	7	14
	pT2	12	24
	pT3	21	42
	pT4	10	20
DOI	≤5 mm	7	14
	5–10 mm	12	24
	>10 mm	31	62

**Table 2:** Comparison of surgeon–pathologist communication methods.

	Traditional Method	Digitalized Method
Data handling	- traditional labeling with limited visualization - potential for loss of important details due to the complex structure and irregular surface	- comprehensive 3D models preserve all details - allows thorough review and precise measurement - facilitates better diagnosis and treatment planning - enhances multidisciplinary discussions
Specimen annotations	- hand-drawn diagrams or simple annotations - higher risk of miscommunication due to complex anatomy - difficulty in visualizing intricate details. - marking with ink or thread; physical specimen handling for examination - limited ability to revisit original specimen post-sectioning - difficult to communicate complex margins	- high-resolution 3D scans capturing complex anatomy accurately - enhances communication with clear and detailed visuals - facilitates precise identification of tumor margins - Virtual 3D models with detailed annotations - reduces handling - provides a permanent record that can be re-examined anytime - enhances the ability to review and share complex margin information
Data storage	- use of thread or beads to mark margins - manual annotations on paper or report - potential loss of spatial orientation post-sectioning - risk of annotation errors due to irregular surfaces	- 3D scanned models with precise digital annotations - easy to visualize and review; accurate spatial orientation maintained - annotations are directly applied to the 3D model, reducing errors

## Data Availability

The data are available within this article. Original raw data are available upon request to the corresponding author.
